# Correction: The architecture of invaginating rod synapses slows glutamate diffusion and shapes synaptic responses

**DOI:** 10.1085/jgp.20241374607242025C

**Published:** 2025-07-30

**Authors:** Wallace B. Thoreson, Thomas M. Bartol, Nicholas H. Conoan, Jeffrey S. Diamond

Vol. 157, No. 3 | https://doi.org/10.1085/jgp.202413746 | February 28, 2025

*JGP* regrets that, because of a text conversion error, the units of measure in Table 1 were presented as millimeters instead of microns. The corrected version of Table 1 appears here, with the measurements in bold. The error appears in print and in PDFs downloaded before July 24, 2025.

**Table 1.**
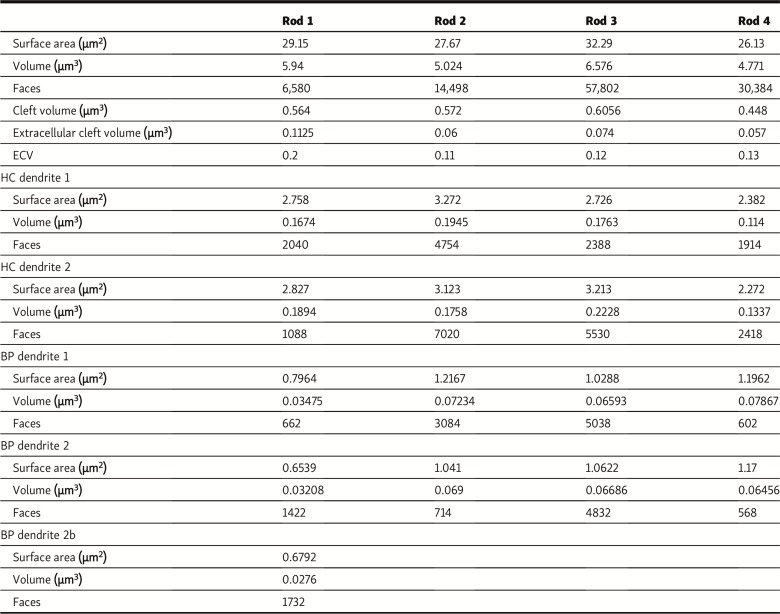
Details of mesh structures of rods, HC dendrites, and RBP dendrites used for simulations

